# Prediction of COVID-19 Hospitalization and Mortality Using Artificial Intelligence

**DOI:** 10.3390/healthcare12171694

**Published:** 2024-08-26

**Authors:** Marwah Ahmed Halwani, Manal Ahmed Halwani

**Affiliations:** 1 College of Business, King Abdulaziz University, Rabigh 21589, Saudi Arabia; 2 Emergency Department, College of Medicine, King Abdulaziz University, Jeddah 21589, Saudi Arabia; mahalawani@kau.edu.sa

**Keywords:** artificial intelligence, clinical decision support systems, predictive tools, disease severity, mortality

## Abstract

Background: COVID-19 has had a substantial influence on healthcare systems, requiring early prognosis for innovative therapies and optimal results, especially in individuals with comorbidities. AI systems have been used by healthcare practitioners for investigating, anticipating, and predicting diseases, through means including medication development, clinical trial analysis, and pandemic forecasting. This study proposes the use of AI to predict disease severity in terms of hospital mortality among COVID-19 patients. Methods: A cross-sectional study was conducted at King Abdulaziz University, Saudi Arabia. Data were cleaned by encoding categorical variables and replacing missing quantitative values with their mean. The outcome variable, hospital mortality, was labeled as death = 0 or survival = 1, with all baseline investigations, clinical symptoms, and laboratory findings used as predictors. Decision trees, SVM, and random forest algorithms were employed. The training process included splitting the data set into training and testing sets, performing 5-fold cross-validation to tune hyperparameters, and evaluating performance on the test set using accuracy. Results: The study assessed the predictive accuracy of outcomes and mortality for COVID-19 patients based on factors such as CRP, LDH, Ferritin, ALP, Bilirubin, D-Dimers, and hospital stay (*p*-value ≤ 0.05). The analysis revealed that hospital stay, D-Dimers, ALP, Bilirubin, LDH, CRP, and Ferritin significantly influenced hospital mortality (*p* ≤ 0.0001). The results demonstrated high predictive accuracy, with decision trees achieving 76%, random forest 80%, and support vector machines (SVMs) 82%. Conclusions: Artificial intelligence is a tool crucial for identifying early coronavirus infections and monitoring patient conditions. It improves treatment consistency and decision-making via the development of algorithms.

## 1. Introduction

A virus is an infectious microbe with a unique genome and protein layer that can reproduce within live cells. By hijacking host cells, these tiny, potent viruses can cause significant health issues [1]. SARS-CoV-2, a new coronavirus, belongs to a larger family of pathogenic viruses that target the respiratory system of humans. It was discovered in 2002 and caused mild infection in China [2]. The seventh strain of SARS-CoV-2, COVID-19, emerged in December 2019, causing respiratory problems and having high transmission rates among species [3]. COVID-19, induced by SARS-CoV-2, has resulted in widespread morbidity and mortality [4]. Despite immunizations, there is a need to prevent morbidity and death from severe COVID-19, especially among vulnerable groups [5]. Evidence points to a vicious loop of immunological dysfunction, endothelial damage, complement activation, and microangiopathy, making these processes critical [6].

In January 2020, the WHO labeled it a public health emergency of international concern (PHEIC) because of its lethal effect on human life [7]. The World Health Organization (WHO) proclaimed COVID-19 a worldwide pandemic on 11 March 2020 [8]. COVID-19 swept over the world in 2020, infecting over 623 million people and causing over 6 million fatalities globally, as well as more than 5 million hospitalizations in the United States by 1 September 2022 [9]. Pandemics and epidemics are characterized by the spread of infectious diseases over a specific period, leading to significant morbidities and mortalities. The SARS epidemic, which infected over 8096 individuals and resulted in over 770 deaths, had greatly devastating effects [10]. Over 213 nations and territories have been affected by the pandemic since its first outbreak in China, infecting more than 98,529,820 people and killing more than 2,116,101 people. The World Health Organization has declared COVID-19 a pandemic, and experts are formulating measures to mitigate its impact on human health and the economy [11].

COVID-19 has a substantial impact on healthcare systems, particularly in patients with acute respiratory syndrome (ARS), necessitating early prognosis for innovative therapies and better results, especially in those with comorbidities [12]. RT-PCR is the standard method for detecting COVID-19 patients as early as possible for effective therapy and containment [13]. Advances in alternative diagnostic technologies are required to speed up detection and treatment, as healthcare professionals and medical personnel are limited, leading to radiologists’ becoming overburdened [14]. In conjunction with COVID-19-related outcomes, the scientific community has widely supported artificial intelligence (AI), a concept encompassing computer systems capable of completing tasks that would otherwise require human intelligence [15].

AI specialists recommend creating ML and DL approaches to help radiologists diagnose pneumonia using imaging modalities and chest scans, which would enable physicians to better combat the disease [16,17]. Using computer algorithms to discover data regularities and categories them, ML is an AI branch with the potential for achieving high prediction accuracy and scalability, especially in fast-paced scenarios like the COVID-19 pandemic, which requires models that can adapt to changing data sources [18].

Classification and regression accuracy are improved with deep learning approaches because the latter have autonomous learning and feature representation capabilities, thereby eliminating the need for human expertise [19]. The development of auxiliary tools for detecting COVID-19-infected humans is crucial. Computer Tomography (CT) and chest X-ray (CXR) images of the lungs are linked to COVID-19 detection [20]. AI systems have been used by healthcare practitioners since 1976 for investigating, anticipating, and predicting diseases, including medication development, clinical trial analysis, and pandemic forecasting [21].

Considering the continually altering COVID-19 due to vaccination and viral mutations, there is an unmet clinical need for a prediction tool based on robust characteristics. Despite advancements in COVID-19 detection, there is no risk prediction model for early disease severity identification. Recent models and artificial networks have high sensitivity and specificity for predicting morbidity and mortality, but they rely on genetic susceptibility, requiring screening for multiple mutations that do not apply to the general population. The current study develops a risk prediction model for COVID-19 outcomes using artificial networks and minimal routine laboratory indices, focusing on admission to the Emergency Department to enhance its value in clinical practice.

## 2. Literature Review

Globally, about 25 million COVID-19 fatalities have been documented, and patients may require intensive care for up to four weeks, which puts a strain on healthcare systems. Prediction models can help clinical decision-making. A study conducted by Sharma et al. in 2020 examines the prediction of COVID-19 using machine learning and big data, taking into account all important factors. It was discovered that some algorithms have weak prediction patterns, resulting in inverted anticipated values. From 30 January to 30 May 2020, the study used two classification methods for Indian COVID-19 cases, as well as a population index. The Bayes point machine and logistic regression algorithms achieved the highest accuracy of 99.6% and 99.4%, respectively. The findings imply that anticipating future COVID-19 fatalities can aid in medical decision-making, particularly when immediate treatment is required [22].

A retrospective cohort analysis by Guan X et al., in 2021, of 1270 COVID-19 patients discovered that six major predictors of death were disease severity, age, high-sensitivity C-reactive protein (hs-CRP), lactate dehydrogenase (LDH), Ferritin, and interleukin-10. The simple-tree XGBoost model, which incorporated these characteristics, predicted death risk with over 90% accuracy and 85% sensitivity, with F1 scores more than 0.90 in both training and validation datasets. These findings might be useful in identifying high-risk situations [23]. The COVID-19 pandemic has raised worldwide healthcare demand, needing timely clinical evaluation. Using clinical data such as lymphocyte count, LDH, and CRP, Yan et al. predicted COVID-19 mortality with 90% accuracy. High LDH levels signal a need for emergency medical intervention. This offers a rule for prioritizing high-risk patients [24].

Supervised learning algorithms have been widely used in predicting COVID-19 results. Studies have been demonstrated on clinical data such as demographics, comorbidities, and test findings. These models can predict hospitalization and mortality risks with high accuracy. Maghdid et al. used a CNN-based model to analyze chest X-rays and CT images, reaching high prediction accuracy for severe COVID-19 patients [25]. The study based on generative adversarial networks (GANs) offers a data-efficient deep network for detecting COVID-19 on CT images. This technology makes more CT scans available while also estimating the parameters of convolutional and fully linked layers using synthetic and augmented data. The GAN-based deep learning model outperforms conventional models for COVID-19 detection, with ResNet-18 and MobileNetV2 performing best on the COVID-19 and Mosmed datasets, respectively [26]. Wynants and colleagues examined 145 models for COVID-19 prognosis, including 23 that predicted death. They discovered significant bias, imprecise reporting, and no external validation. As a result, the employment of these anticipated models is not encouraged in current practice [27].

COVID-19 has resulted in the prevalence of low-quality clinical prediction models. More actions are needed to serve patients in all areas of healthcare by building model development frameworks. The potential of AI in predicting COVID-19 hospitalization and mortality is intriguing, but issues with data quality, model interpretability, and generalizability must be solved before it can be fully utilized.

## 3. Materials and Methods

Research Ethics Committee boards approved a study, waived written informed consent, and de-identified patient data to avoid confidentiality breaches.

Patient cohorts: A cross-sectional study was conducted after approval from the Research Ethics Committee of King Abdulaziz University (KAU), Saudi Arabia. The study used sequential sampling approaches to include 50 Real-Time Polymerase Chain Reaction (RT-PCR)-positive COVID-19 patients from KAU’s coronavirus isolation wards. Medical records were collected and analyzed by clinical teams. The results of RT-PCR were obtained from electronic medical records using approved TaqMan One-Step Kits. Positive results on the last-performed test confirmed diagnosis for patients with multiple assays.

Demographic and clinical information: Demographic information about each patient was gathered, including age, gender, symptoms, white blood cell and lymphocyte counts, comorbidity status, and history of COVID-19 exposure. Information on patients’ mechanical breathing, intense medical treatment, death progression, admission and discharge times, and illness severity were all recorded based on symptom records, clinical findings, and chest X-rays. A pre-designed form was used to record each patient’s demographic information, including age and gender, signs and symptoms, illness severity (mild, moderate, severe), and laboratory findings. Furthermore, the length of the hospital stay and the outcome, whether the patient recovered or died, were reported. Treatment information and clinical results were tracked over the following weeks until discharge (Table S1).

Predictive analysis: Predictive analytics, a subset of advanced analytics, uses historical data, statistical algorithms, and machine learning techniques to forecast future occurrences or outcomes. Through the examination of data patterns, trends are identified, and future behavior or events are predicted. Historical data serve as the basis for training forecasting models in this area. These models are then used to extrapolate predictions from new or unpublished data. Predictions range from simple binary outcomes such as positive or negative responses to complex scenarios involving multiple possible outcomes. In the current study, the steps outlined in the following paragraphs were followed to predict disease severity in terms of hospital mortality among COVID-19 patients. The study recorded demographic details, signs and symptoms, disease severity (Table 1), as well as laboratory findings such as Bilirubin, AST, ALT, phosphomonoesterases, GGT, protein, CRP, D-Dimers, white blood cells, platelets, LDH, prothrombin time, and Ferritin (ng/mL) (Table 2).

Data preprocessing:

a. Data cleaning and transformation: The data were cleaned through the handling of missing values. Missing values in the dataset were handled by using a boxplot. Records lacking essential data points were excluded from the analysis to maintain the models’ integrity. The categorical variables were coded according to categorical variables, and the quantitative variables’ missing values were replaced by their mean. The outcome variable (hospital mortality) was properly labeled as death = 0 or survival = 1. All the baseline investigations, clinical symptoms, and laboratory findings were labeled as predictors.
b. Dataset splitting: The data were divided into training and testing sets, with the training set used for model development and the testing set reserved for performance evaluation. To optimize the models’ hyperparameters and enhance generalizability, a 5-fold cross-validation technique was applied. This approach helps minimize variance and bias in the models’ performance.

Machine learning algorithms:

The algorithms used in the study were decision trees, SVM, and random forest.

Hyperparameters:

a. Decision trees: The model’s hyperparameters include a maximum depth of 10 and a minimum sample split of 2. The criterion used for measuring the quality of splits is Gini impurity.
b. Support vector machines (SVMs): The model used a radial basis function (RBF) kernel, which is effective in high-dimensional spaces. The regularization parameter was set to 1.0, balancing the trade-off between maximizing the margin and minimizing classification errors. The kernel coefficient \(\gamma\) was set to ‘scale’. This helps in capturing the non-linear relationships in the data. The tolerance for stopping criteria was set to 0.001. A 5-fold cross-validation was performed to ensure robustness and prevent overfitting.
c. Random forest: The model used 100 trees, balancing computational efficiency and model performance. The maximum depth of each tree was set to none, allowing trees to grow until all leaves were pure or until all leaves contained less than the minimum samples required to split. The minimum number of samples required to split an internal node was set to 2. The model used the Gini impurity criterion to measure the quality of a split. Bootstrap samples were used when building trees to reduce overfitting. A 5-fold cross-validation was performed to tune the hyperparameters and validate the model’s performance.

These hyperparameters were optimized to enhance the predictive accuracy of the SVM and random forest models in predicting COVID-19 patient mortality.

Training process:

The dataset is divided into training and testing sets, typically with an 80–20 split. Cross-validation, such as 5-fold cross-validation, is performed to tune hyperparameters and prevent overfitting. The model is then trained using the training set and validated using the validation set. Finally, the model’s performance is evaluated on the test set using appropriate metrics, such as accuracy.

Technical characteristics of computer used:

The computer utilized for the analysis is equipped with an Intel Core i7-9700K CPU, 32 GB DDR4 RAM, and an NVIDIA GeForce RTX 2080 Ti GPU. It also features 1 TB of SSD storage and runs on the Windows 10 Pro operating system. The software environment includes Python 3.8 as the programming language, with libraries such as Scikit-learn 0.24.2 for machine learning algorithms, Pandas 1.2.4 for data manipulation, NumPy 1.20.2 for numerical computations, and Matplotlib 3.4.2 and Seaborn 0.11.1 for data visualization. The analysis is conducted using the Jupyter Notebook 6.3.0 integrated development environment (IDE).

**Figure 1:**
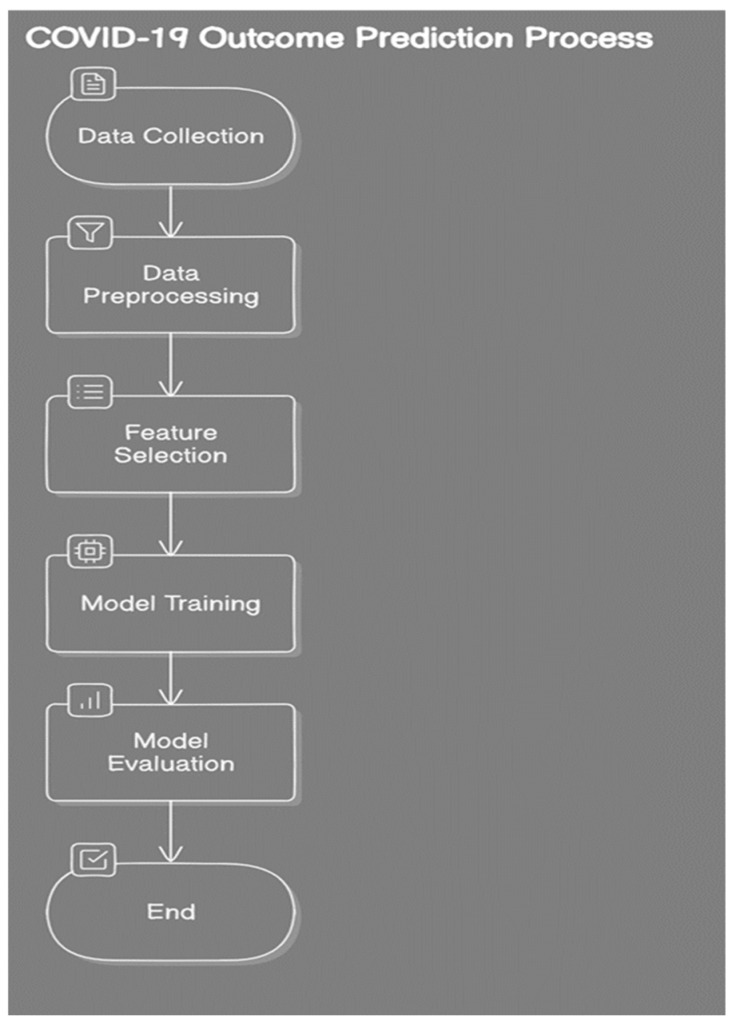
Block diagram of the study.

Block diagram:

The study follows a structured approach consisting of several key steps. First, data collection involves gathering patient data, including demographics, symptoms, and laboratory results. Second, data preprocessing entails cleaning and preparing the data for analysis. Third, feature selection identifies the key features that impact the prediction of COVID-19 outcomes. Fourth, model training is performed using the selected features to train machine learning models. Fifth, model evaluation assesses the models’ performance using accuracy, precision, and recall metrics. Finally, the prediction phase involves using the trained models to predict outcomes for new patients (Figure 1).

Statistical analysis: The data were entered and analyzed in SPSS. Mean ± standard deviation (SD) was calculated for quantitative variables and frequency/percentages for qualitative variables. The mean difference among laboratory findings for the outcome variables was calculated through an independent sample *t*-test. *p*-value < 0.05 was significant.

## 4. Results

### 4.1. Demographics and Baselines of COVID-19 Patients

The study included 50 patients, with an average age of 50.9 years (SD = 15.09). Patients stayed in the hospital for an average duration of 14.6 days (SD = 2.8). Gender distribution revealed 56.0% male and 44.0% female participants. Disease severity varied, with 34.0% experiencing mild symptoms, 46.0% moderate, 14.0% severe, and 6.0% critical conditions. Common symptoms included fever (48.0%), fatigue (38.0%), cough (36.0%), sore throat (24.0%), and diarrhea (24.0%). Less common symptoms were nausea (16.0%) and abdominal pain (10.0%). The majority of patients (88.0%) survived, while 12.0% unfortunately died due to COVID-19 (Table 1).

### 4.2. Laboratory Parameters in COVID-19 Patients

The analysis of laboratory parameters in the COVID-19 patients revealed significant details. The average white blood cell count was 11.91 × 10^9^/L, indicating a broad range, predominantly above the normal threshold. The platelet count averaged 220.0 × 10^9^/L, remaining within the expected range. However, the C-reactive protein (CRP) levels were notably elevated, averaging 60.18 mg/L, suggesting heightened inflammation. The lactate dehydrogenase (LDH) levels exhibited a mean of 296.98 U/L, indicating potential tissue damage. The Ferritin levels were also elevated, with a mean of 479.89 ng/mL, implying inflammation or iron overload. The D-Dimer levels showed an average of 438.59 mg/L, indicative of possible blood clot formation. While alkaline phosphatase (ALP), gamma-glutamyl transferase (GGT), alanine transaminase (ALT), and aspartate aminotransferase (AST) levels generally fell within normal ranges, the Bilirubin levels were slightly elevated, averaging 0.63 mg/dL. The prothrombin time and calcium levels remained within the expected parameters, while the potassium levels averaged 4.05 mEq/L, within normal limits (Table 2). There was a significant difference in CRP, LDH, Ferritin, ALP, Bilirubin, D-Dimers, and hospital stay, with a *p*-value < 0.05 (Table 3).

### 4.3. Prediction of Mortality

**Figure 2:**
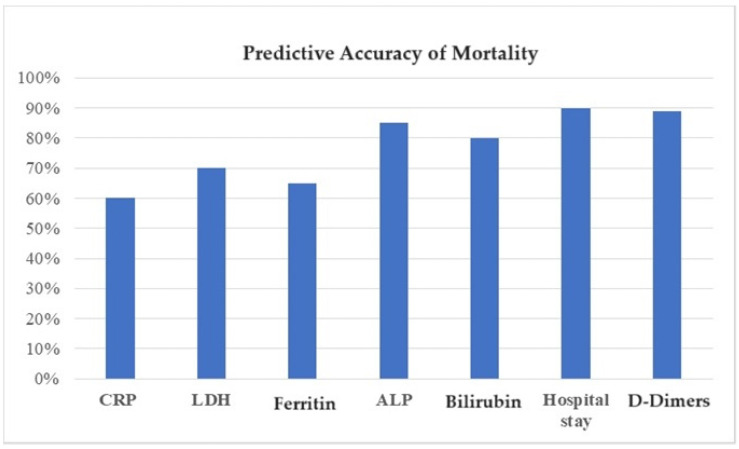
Predictive accuracy of mortality according to lab findings.

The hospital stay, D-Dimers, ALP, Bilirubin, LDH, CRP, and Ferritin levels were higher in COVID-19 patients indicated in Figure 2.

Increased levels indicated its association with mortality. The algorithm’s accuracy was calculated and indicated high accuracy of the decision tree at 76%, random forest 80%, and SVM 82%; the decision tree was calculated, indicating a high decision tree (Table 4).

### 4.4. Hypothetical Confusion Matrix for SVM

Table 5 shows that 41 patients survived, while 42 did not. The performance metrics of the model are as follows: sensitivity was 83.67%, specificity was 82.35%, positive predictive value (PPV) was 82.0%, negative predictive value (NPV) was 84.0%, and overall accuracy was 83%.

The formula used to evaluate the diagnostic accuracy:

$$Accuracy=\frac{TP+TN\;\;\;}{TP+TN+FP+FN}$$

## 5. Discussion

The research included 50 patients with various illness severities, with the majority feeling fever, weariness, cough, sore throat, and diarrhea. The majority survived, with 56.0% males. The research of COVID-19 patients revealed laboratory measures, including an average white blood cell count that was higher than normal, a platelet count that was within the predicted range, raised C-reactive protein levels, probable tissue damage, ferritin levels, and D-Dimer levels. Other indicators, including alkaline phosphatase, gamma-glutamyl transferase, alanine transaminase, and aspartate aminotransferase, were typically within normal limits. Bilirubin levels were slightly higher, but prothrombin time, calcium, and potassium levels were within normal ranges.

The study conducted by Yaşar Ş et al. [28] demonstrates that, by utilizing AI, the prognosis of COVID-19 patients is mostly based on clinical characteristics such as vital signs and laboratory testing, which is also indicated in our work. The shortcoming of the previous study was that they did not use X-rays as a prediction for COVID-19 severity; this is also the limitation of our study. The work also emphasizes the feasibility of combining clinical information and laboratory values in a single system, offering a fresh viewpoint on prognostic AI systems. Acute respiratory distress syndrome affects 15% of patients, and more than half of ICU admissions are due to hypoxia or respiratory fatigue. Analysis using AI systems based on clinical data can predict disease development more accurately than clinical data alone, improving patient care by combining information from different sources [29]. The current study also emphasized the use of AI-based clinical prediction for the severity of COVID-19 to make it a predictive tool.

Early detection and treatment of COVID-19 disease is crucial for decreased mortality, especially for severely ill patients. Previous research using imaging data from COVID-19 patients has mostly focused on diagnosis rather than prognosis [30]. Prognostic models may forecast mortality, morbidity, and other outcomes, and they have real-world applications in patient identification, bed management, situational awareness, and resource allocation [31].

Computers are expected to play a crucial role in combating global health emergencies, with AI being extensively applied to predict clinical outcomes of hospitalization and mortality. AI is produced by computer systems capable of doing tasks that require human-like intellect, with machine learning playing a critical role in providing high prediction accuracy and scalability [32]. Substantial efforts from the scientific community have aimed to integrate AI, particularly machine learning, into predictive modeling for COVID-19-related outcomes [33]. ML and deep learning (DL) are key components of AI that use algorithms to learn and adapt from data. DL, a subset of machine learning, extracts complicated information using neural networks with numerous layers; it includes deep, deep belief, and recurrent learning [34]. This research introduced predicting COVID-19 diagnosis based on baseline demographics, comorbidities, vital signs, and lab findings. Predictive models can be used for diagnosis when the testing capacity is restricted, or they can be combined with clinical judgment. They uncover crucial clinical characteristics associated with positive diagnosis, giving information for effective patient stratification and population screening. The single-tree model’s decision algorithm can be used in healthcare settings. The studies indicated acute respiratory distress syndrome (ARDS) and/or sepsis are strong markers of a positive COVID-19 diagnosis [35].

ML algorithms were associated with a positive COVID-19 diagnosis in both symptomatic and asymptomatic patients. Four models indicated age, lab results, comorbidities, vital signs, and hematologic characteristics as predictors of a positive diagnosis. Abnormal liver function tests, as well as low white blood cell count and hemoglobin levels, have previously been identified as indications of COVID-19 severity. These data may help predict the severity of COVID-19 [36]. The study’s innovative use of machine learning classification may face significant challenges in model interpretability, which is essential for effective clinical decision-making. The complexity of these models can obscure the reasoning behind predictions. Moreover, by concentrating on comorbidities and their interactions with symptoms, the study may neglect other crucial factors, such as mental health, social determinants of health, and patient behavior, which also play a key role in COVID-19 outcomes.

Our results discovered that blood CRP, LDH, Ferritin, ALP, Bilirubin, and D-Dimer levels were the strongest predictive characteristic of COVID-19 diagnosis, which is consistent with earlier research identifying serum levels as a biomarker of clinical severity and poor prognosis. Numerous research has investigated the significance of biochemical and hematological indicators in COVID-19 to develop an algorithm for identifying poor prognosis, ventilation, and early intervention. Despite this, there is little agreement on this subject, and future studies should focus on regional biomarker profiles.

A comprehensive overview in a study conducted in 2021 found AI applications in the field of COVID-19 address various areas and have many benefits. In disease diagnosis, AI helps in the interpretation of various tests and symptoms and facilitates the rapid and accurate identification of infections. AI also contributes to patient monitoring by enabling continuous assessment and timely intervention. It plays a crucial role in determining the severity of a patient’s condition and helps healthcare providers prioritize treatment strategies effectively. When processing imaging tests related to COVID-19, AI algorithms improve the analysis of radiological scans and enable the rapid detection of abnormalities indicative of infection by the virus. Epidemiology benefits from AI-driven predictive modeling, which helps to predict outbreaks, track trans-mission patterns, and develop targeted intervention strategies [37]. However, this paper’s case studies may not be diverse enough, restricting a comprehensive understanding of AI’s effectiveness across different healthcare systems. While ethical concerns such as data privacy and algorithmic bias are acknowledged, they are not thoroughly examined. Moreover, although the paper addresses emerging technologies and policy recommendations, it falls short of providing specific examples or actionable steps for AI implementation after the pandemic.

A deep learning system has been developed to predict the malignant progression of COVID-19 using clinical data and CT scans studied in 2020 in China. The system achieved an average AUC of 0.874 in a multicenter study. The system automatically identifies key indicators contributing to malignant progression, including Troponin, Brain natriuretic peptide, White cell count, Aspartate aminotransferase, Creatinine, and Hypersensitive C-reactive protein [38]. Another important study in 2020 conducted by Wynants et al. provided a detailed assessment of COVID-19 diagnosis and prognosis, assessing prediction models’ accuracy and value in detecting suspected infections, forecasting patient outcomes, and identifying persons at increased risk of infection or hospitalization [39].

AI is currently being used to predict COVID-19 mortality and hospitalization by combining patient demographics, medical history, vital signs, and laboratory data. The objective is to identify high-risk individuals so that they can receive prompt medical treatment. Mortality studies employ comparable input factors, with an emphasis on illness severity and progression. Machine learning also predicts hospitalization and death, taking into account the interplay of these events [40].

Due to their excellent accuracy, machine learning algorithms, notably random forest, have been successful in predicting COVID-19-related hospitalization and mortality. Random forest operates by constructing multiple decision trees and aggregating predictions, effectively capturing complex data relationships [41]. Its versatility allows for handling diverse input variables without extensive pre-processing. Additionally, random forest provides insights into feature importance, aiding in identifying key predictors of COVID-19 outcomes. These analytical advantages make random forest a valuable tool in medical research and decision-making processes surrounding COVID-19 [42]. The study revealed the efficacy of predictive models in COVID-19 diagnosis, allowing for effective screening and patient classification. This is critical given the current pandemic’s impact on huge populations, which necessitates more efficient testing resource allocation and improved patient care.

Another study examined clinical features and lab indicators in severe and non-severe COVID-19 patients, identifying significant differences in neutrophil-to-lymphocyte ratio, C-reactive protein, and lactate dehydrogenase. They developed a decision tree model that accurately predicted mortality in critically ill patients with 98% precision, helping prioritize treatment for high-risk individuals [43]. These findings were also comparable with our study, which also indicates that the tree predicts COVID mortality with good precision. However, a major shortcoming is the difficulty in generalizing AI models to different populations and settings. Models trained on specific datasets may not perform accurately when applied to new or diverse groups, leading to unreliable predictions.

Joaquim Carreras’ study employed artificial intelligence (AI) to analyze celiac disease using a transcriptomic panel focused on autoimmune discovery. The AI models demonstrated exceptional accuracy, ranging from 95% to 100%, in predicting celiac disease based on the autoimmune gene panel. This highlights the models’ effectiveness in distinguishing celiac disease patients from control subjects [44].

## 6. Conclusions

The gold-standard PCR test for COVID-19 is constrained by high turnaround times, a lack of specialized equipment, and low sensitivity, providing a challenge to global healthcare systems. NHS guidelines require testing of all emergency admissions, regardless of clinical suspicion, emphasizing the critical requirement for prompt and accurate COVID-19 exclusion in acute care settings. Our models have a strong predictive performance, making them suitable for screening COVID-19 diagnoses in emergency rooms. They help make rapid treatment decisions, guide safe patient streaming, and act as a pre-test for diagnostic molecular testing. Key benefit categories include viral-free individuals who were properly predicted to be COVID-19-negative. This strategy is extensively used in clinical practice. The clinically focused approach ruled out COVID-19 in enriched subpopulations that were more likely to test positive, proposing conclusive testing, comparable to the D-Dimer test for suspected deep-vein thrombosis and pulmonary embolism.

The integration of AI has significantly advanced the fight against COVID-19. From diagnosis to predicting outcomes to modeling future trends, AI has played a crucial role in interpreting data, improving patient care, and predicting outbreak dynamics. In addition, the application of ML models has significantly improved predictive accuracy and provided valuable insights into COVID-19-related hospital admissions and mortality rates. During a global health crisis, AI can improve public health and solve pandemic-related issues by improving decision-making and patient outcomes.

Until now, early detection models have mostly focused on radiological imaging evaluation. Few studies have evaluated routine laboratory tests, with studies to date including small numbers of patients with confirmed COVID-19, using PCR results for data labeling, and thus not ensuring disease freedom in so-called negative patients, as well as not being validated in the clinical population that is the target for their intended use.

## 7. Limitations of the Study

The use of small control cohorts during training is a shortcoming of this study since it fails to expose models to the breadth and range of alternate infectious and non-infectious diseases, including seasonal pathologies. Furthermore, while the application of artificial intelligence approaches for early detection has enormous potential, several published models are highly biased.

## Figures and Tables

**Table 1:** COVID-19 patients’ demographics and baseline characteristics.

Variables		
Age (Mean ± SD)	50.9 ± 15.09	
Hospital Stay (Days)	14.6 ± 2.8	
	Frequency	Percentages (%)
Gender		
Male	28	56.0
Female	22	44.0
Disease Severity		
Mild	17	34.0
Moderate	23	46.0
Severe	7	14.0
Critical	3	6.0
Sign and Symptoms		
Fever	24	48.0
Cough	18	36.0
Sore throat	12	24.0
Diarrhea	12	24.0
Fatigue	19	38.0
Nausea	8	16.0
Abdominal pain	5	10.0
Outcome		
Death	6	12.0
Survived	44	88.0

**Table 2:** Baseline laboratory.

Laboratory Parameters	Normal Range	Mean ± SD	Minimum	Maximum	Range
White blood cell × 10^9^/L	3.5–9.5	11.91 ± 12.9	0.741	76.6	75.85
Platelets × 10^9^/L	125–350	220.0 ± 80.5	40.0	418.0	378.0
CRP (mg/L)	<3	60.18 ± 83.01	0.10	322.13	322.03
LDH (U/L)	140 to 280	296.98 ± 163.01	155.0	1044.0	889.0
Ferritin (ng/mL)	12 to 300	479.89 ± 436.07	8.0	1675	1667
D-Dimers (mg/L)	>0.5	438.59 ± 443.0	0.2	1600.0	1599.8
Alkaline phosphatase (ALP), (U/L)	44–147	85.12 ± 23.64	40.0	135.00	95.0
Gamma-glutamyl transferase (GGT), (U/L)	0–30	40.12 ± 16.54	10.0	79.0	69.0
Alanine transaminase (ALT), (U/L)	7–50	33.28 ± 11.12	17.0	60.0	43.0
Aspartate aminotransferase (AST), (U/L)	15–40	38.64 ± 13.93	18.0	75.0	57.0
Bilirubin (mg/dL)	<0.3	0.63 ± 0.32	0.2	1.4	1.2
Prothrombin time/sec	10–13/sec	11.6 ± 1.47	8.0	14.0	6.0
Calcium (mg/dL)	8.5 to 10.2	8.8 ± 0.33	8.0	9.6	1.6
Potassium (mEq/L)	3.5–5	4.05 ± 0.80	2.9	8.8	5.9

**Table 3:** Mean difference of laboratory findings among outcome variables (survival/death).

Laboratory Findings	Outcome	Mean ± SD	*p*-Value
WCC	Survival	10.81 ± 9.34	0.104
	Death	19.99 ± 28.37	
PLT	Survival	222.25 ± 72.39	0.605
	Death	203.83 ± 134.95	
CRP	Survival	51.17 ± 69.86	≤0.05 *
	Death	124.80 ± 139.48	
LDH	Survival	271.52 ± 102.10	≤0.001 **
	Death	483.67 ± 351.06	
Ferritin	Survival	439.42 ± 365.26	≤0.05 *
	Death	835.98 ± 819.24	
D-Dimers	Survival	332.47395 ± 345.07	≤0.001 **
	Death	1216.8 ± 271.52	
ALP	Survival	81.73 ± 22.25	≤0.001 **
	Death	110.00 ± 19.48	
GGT	Survival	38.80 ± 16.88	0.127
	Death	49.83 ± 10.21	
ALT	Survival	32.68 ± 11.53	0.308
	Death	37.67 ± 6.53	
AST	Survival	37.70 ± 14.41	0.202
	Death	45.50 ± 7.31	
Bilirubin	Survival	0.60 ± 0.30	≤0.05 *
	Death	0.88 ± 0.39	
Prothrombin time	Survival	11.64 ± 1.40	0.641
	Death	11.33 ± 2.07	
Calcium	Survival	8.81 ± 0.35	0.595
	Death	8.73 ± 0.23	
Potassium	Survival	4.07 ± 0.83	0.665
	Death	3.92 ± 0.62	
Hospital stay	Survival	14.57 ± 2.96	≤0.001 **
	Death	23.00 ± 2.83	

*p*-value ≤ 0.05 * significant, *p*-value ≤ 0.01 ** strongly significant, results from independent sample *t*-test.

**Table 4:** Predictive accuracy of algorithms.

Algorithms	Accuracy (%)
Decision tree	76%
Random forest	80%
SVM	82%

**Table 5:** Hypothetical confusion matrix for SVM.

Actual Findings	Results from SVM	
	Positive (Survived)	Negative (Died)
Positive (survived)	41	9
Negative (died)	8	42
Sensitivity	83.67%	
Specificity	82.35%	
Positive predicted value (PP V)	82.0%	
Negative predictive value (NPV)	84.0%	
Accuracy	83.0%	

## Data Availability

Data supporting reported results can be found, including links to publicly archived datasets analyzed or generated during the study.

## Supplementary Materials

The following supporting information can be downloaded at: https://www.mdpi.com/article/10.3390/healthcare12171694/s1. Table S1: COVID data.
